# Supplementary material for: Validation of polymorphic Gompertzian model of cancer through in vitro and in vivo data
Source: PLoS One. 2025 Jan 9;20(1):e0310844. doi: 10.1371/journal.pone.0310844 (PMC11717199; doi:10.1371/journal.pone.0310844)
Supplement: S1 Appendix — (PDF) [file pone.0310844.s001.pdf]

S1 Appendix.
Procedures of fitting the polymorphic Gompertzian model to *in vitro* and *in vivo* data

We fitted the polymorphic Gompertzian model to the *in vitro* and *in vivo* data on cancer population dynamics using GEKKO package. GEKKO is a Python package for machine learning and optimization that allows to dynamically estimate parameters of the differential equations through nonlinear optimization [1].

In the *in vitro* case, we excluded the first three measurements, as they demonstrate fluctuations attributed to the initial stabilization of the system. As the fitting procedure is sensitive to initial values and search bounds, we set physiological lower bounds for the parameters: the growth rate ρ and treatment sensitivity λ are assumed positive. We set the lower bound of the carrying capacity K at 0.9 of the maximum measured population size in the well. As the carrying capacity should indicate the growth limit, we assumed it to be bigger than the measured population size at all time, but accounted for the possible 10% measurement error. To minimize the probability of finding the local minimum instead of the global one, we implemented the grid search over a range of initial parameters values.

The model was fitted with fixed initial values ($S_{pred}(0) = S_{mes}(0)$, $R_{pred}(0) = R_{mes}(0)$, where S_{mes} and R_{mes} are measured sizes of the sensitive and resistant populations, respectively, and S_{pred} and R_{pred} are sizes of sensitive and resistant populations predicted by the model). We also performed fitting of the model with optimized initial conditions and compared the obtained results. However, we decided to proceed with fixed initial population sizes to keep the number of degrees of freedom low.

The *in vivo* data, unlike the *in vitro* data, does not contain information on the tumor composition. Therefore, initial proportions of sensitive and resistant cells needed to be provided as an input for the optimization procedure. To do that, we implemented an additional grid search for the seeding proportions.

The General Gompertz and General von Bertalanffy models were fitted to the same *in vivo* data, using Python package GEKKO [1]. In the optimization procedure, the MSE between measured tumor volumes and predicted by the model values defined as

$$MSE = \frac{1}{n}\sum_{i=1}^{n}\left(x_{pred}(t_i) - x_{mes}(t_i)\right)^2 \tag{S1.1}$$

is minimized. In Eq (S1.1), $x_{mes}(t_i)$ is measured tumor volume at i-th time point, $x_{pred}(t_i)$ is model-predicted volume at i-th time point, and n is number of time points. The fittings of the General Gompertz and General von Bertalanffy models were performed with the same procedure as for the polymorphic Gompertzian model. For more details on the fitting procedure see GitHub (`https://github.com/SobolevaArina/polymorphic_Gompertzian_model`).

References

1. Beal L, Hill D, Martin R, Hedengren J. GEKKO Optimization Suite. Processes. 2018;6(8):106. doi:10.3390/pr6080106.
