# Supplementary material for: Validation of polymorphic Gompertzian model of cancer through in vitro and in vivo data
Source: PLoS One. 2025 Jan 9;20(1):e0310844. doi: 10.1371/journal.pone.0310844 (PMC11717199; doi:10.1371/journal.pone.0310844)
Supplement: S2 Appendix — (PDF) [file pone.0310844.s002.pdf]

S2 Appendix.
Trend categories in tumor volume dynamics in *in vivo* data

In vivo data of 587 patients was split into five categories based on the observed tumor volume dynamics. For each patient, we calculated a vector of tumor volume differences between measurmets made at time point t_{i+1} and at time point t_i for all i. If the difference is positive, then the tumor volume increases between measurements and vice versa. Based on values in the difference vector, we categorized patient cases into trend groups. Categories, the number of cases in each of them, and criteria for classification of the case into one of the categories are presented in Table S2.1.

Table S2.1. Categories of patients according to the observed trend in tumor volume dynamics, the number of cases in each category and criteria of classification.

**Category**	**Number of cases**	**Criteria**
Growth	98	All differences are positive or the sum of positive differences is at least two times greater than the absolute sum of negative differences
Decline	239	All differences are negative or the absolute sum of negative differences is at least two times greater than the sum of positive differences
Delayed response	65	First non-zero difference value is positive, the difference vector contains a negative value and the last measurement is smaller than the first one
U-shape	81	Negative difference value followed by positive, but not vice versa, fluctuations of 10% excluded
Fluctuate	104	All others
