# Supplementary material for: Validation of polymorphic Gompertzian model of cancer through in vitro and in vivo data
Source: PLoS One. 2025 Jan 9;20(1):e0310844. doi: 10.1371/journal.pone.0310844 (PMC11717199; doi:10.1371/journal.pone.0310844)
Supplement: S3 Appendix — (PDF) [file pone.0310844.s003.pdf]

S3 Appendix.
Comparison of the polymorphic Gompertzian model's fit with the fits of the General Gompertz and General von Bertalanffy models

The fit of the polymorphic Gompertzian model to *in vivo* data was compared to the fit of monomorphic General Gompertz and General von Bertalanffy models fitted with the same procedure. These models previously demonstrated the highest accuracy compared to the other textbook models, when fitted to this dataset [1]. The models' equations are as follows:

General Gompertz:

$$\dot{V}(t) = V^{\mu}(\delta - \gamma \ln V), \tag{S3.1}$$

where V is size of the tumor in mm^3, γ is the maximum net growth rate of cancer population, μ and δ are constants.

General von Bertalanffy:

$$\dot{V}(t) = \alpha V^{\mu} - \beta\, V, \tag{S3.2}$$

where V is size of the tumor in mm^3, α is the birth rate of cancer cells, β is the death rate of cancer cells and μ is a constant.

The General Gompertz and General von Bertalanffy models were fitted to the *in vivo* data using Python package GEKKO [2]. Fig S3.1 demonstrates fits of the three models to the five representative example cases from "Growth", "Decline", "Delayed response", "U-shape" and "Fluctuate" categories. All three models can describe "Growth" and "Decline" categories well with the models' dynamics similar to each other (Rows $1-2$ of Fig S3.1). None of the models is able to capture the "Delayed response" trend. In the "U-shape" category the polymorphic Gompertzian model outperforms both General Gompertz and General von Bertalanffy models.

We also compared errors of the polymorphic Gompertzian, General Gompertz and General von Bertalanffy models. We calculated normalized mean square error $nMSE$, which is a mean squared error divided by square of the largest measured volume in the case. For the polymorphic Gompertzian model:

$$nMSE = \frac{1}{n}\sum_{i=1}^{n}\left(\frac{S_{pred}(t_i) + R_{pred}(t_i) - x_{mes}(t_i)}{x_{mes}^{max}}\right)^2, \tag{S3.3}$$

with $x_{mes}(t_i)$ being the measured tumor volume at the i-th time point, $R_{pred}(t_i)$ and $S_{pred}(t_i)$ the resistant and sensitive population volumes at the i-th time point predicted by the model, x_{mes}^{max} the maximum measured tumor volume and n the number of measurements.

For General Gompertz and General von Bertalanffy models:

$$nMSE = \frac{1}{n}\sum_{i=1}^{n}\left(\frac{x_{pred}(t_i) - x_{mes}(t_i)}{x_{mes}^{max}}\right)^2, \tag{S3.4}$$

where $x_{mes}(t_i)$ - measured tumor volume at i-th time point, $x_{pred}(t_i)$- tumor volume at i-th time point predicted by the model, x_{mes}^{max} - maximum measured tumor volume, n - number of measurements.

Violin plots of the $nMSE$ distribution for the General Gompertz, General von Bertalanffy and the polymorphic Gompertzian models across five trend categories are presented in Fig S3.2. The $nMSE$ of the polymorphic Gompertzian model's fit is lower than the one of the General Gompertz model's fit in the "Growth" and "Decline" categories and similar to the fit of the General von Bertalanffy model. The polymorphic Gompertzian model has a higher accuracy than the other models in the "U-shape" category. Table 1 (main text) shows p-values of t-test between the models' $nMSE$ in trend categories.

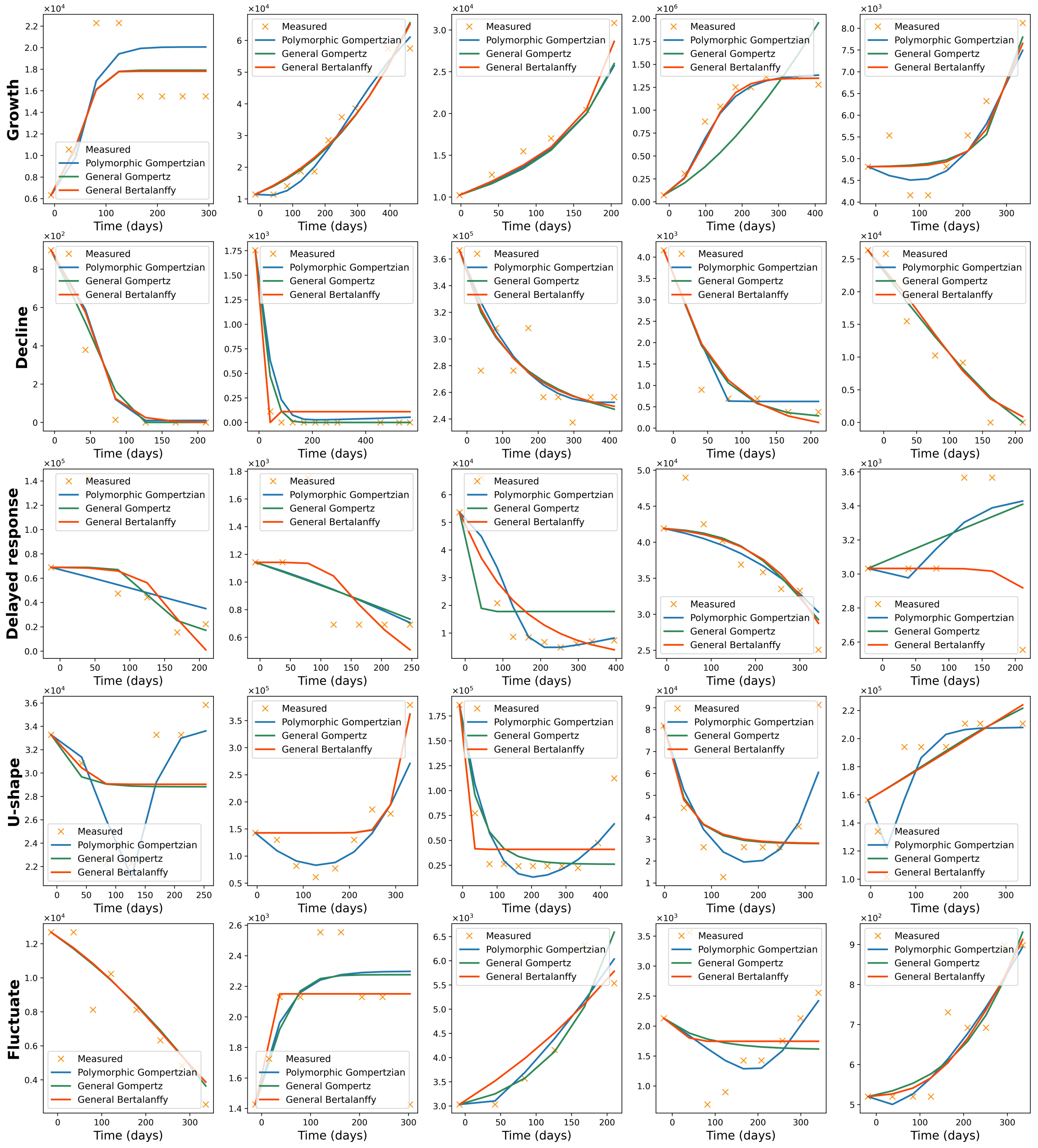

Fig S3.1. Fits of the polymorphic Gompertzian, General Gompertz, and General von Bertalanffy models to *in vivo* data. The models' fits are presented for five patient cases in each trend category: "Growth", "Decline", "Delayed response", "U-shape" and "Fluctuate". Data points are marked with orange crosses. Blue, green, and red lines demonstrate fits of polymorphic Gompertzian, General Gompertz, and General von Bertalanffy models respectively.

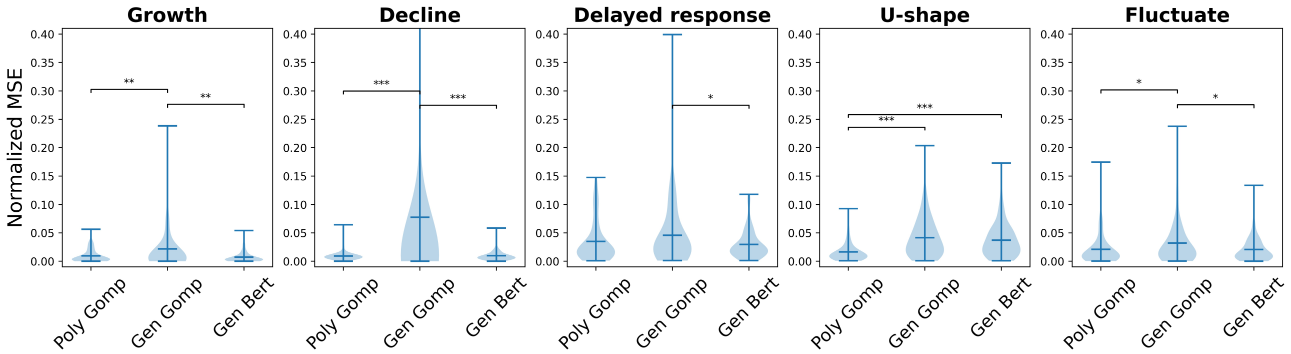

Fig S3.2. Distributions of $nMSE$ of the polymorphic Gompertzian, General Gompertz and General von Bertalanffy models' fits to *in vivo* data across five trend categories. In the "U-shape" category nMSE of the polymorphic Gompertzian model is significantly lower than of the other two models. Stars denote statistically significant differences between means of two groups ($\star$ - p-value < 0.05, $\star\star$ - p-value < 0.01, $\star\star\star$ - p-value < 0.001).

References

1. Ghaffari Laleh N, Loeffler CML, Grajek J, Staňková K, Pearson AT, Muti HS, et al. Classical mathematical models for prediction of response to chemotherapy and immunotherapy. PLOS Computational Biology. 2022;18(2):e1009822. doi:10.1371/journal.pcbi.1009822.

2. Beal L, Hill D, Martin R, Hedengren J. GEKKO Optimization Suite. Processes. 2018;6(8):106. doi:10.3390/pr6080106.
