# Supplementary material for: Validation of polymorphic Gompertzian model of cancer through in vitro and in vivo data
Source: PLoS One. 2025 Jan 9;20(1):e0310844. doi: 10.1371/journal.pone.0310844 (PMC11717199; doi:10.1371/journal.pone.0310844)
Supplement: S4 Appendix — (PDF) [file pone.0310844.s004.pdf]

S4 Appendix.
Parameter values of the polymorphic Gompertzian model across trend categories

We assessed the fitted parameter values of the polymorphic Gompertzian model across trend categories. The distributions of the fitted carrying capacity K, growth rate ρ, treatment sensitivity λ, and the estimated initial proportions of sensitive cells are presented in Fig S4.1. To ensure the statistical significance of the differences between groups, we implemented t-test.

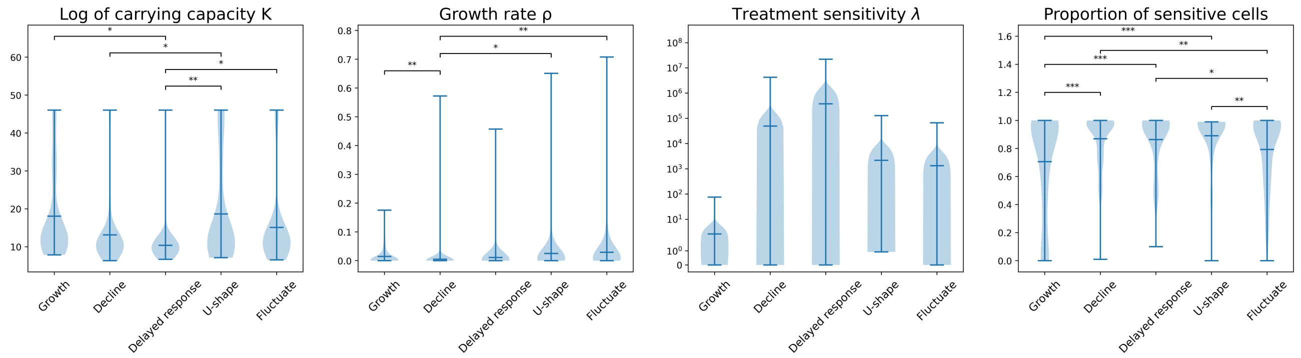

Fig S4.1. Distribution of the polymorphic Gompertzian model's parameter values fitted to *in vivo* data. Violin plots for the logarithmic values of fitted carrying capacity K, growth rate ρ, treatment sensitivity λ and estimated values of initial proportions of sensitive cells of the polymorphic Gompertzian mode's fit to *in vivo* data across five trend categories ("Growth", "Decline", "Delayed response", "U-shape", and "Fluctuate"). Stars denote statistically significant differences between means of two groups ($\star$ - p-value < 0.05, $\star\star$ - p-value < 0.01, $\star\star\star$ - p-value < 0.001).

We observed that the distributions of the model parameters and the initial proportion of sensitive cells across the trend categories are in agreement with the biological features of these groups (Fig S4.1). The carrying capacity captures restrictions of physical space, availability of required nutrients, and also implicitly includes other environmental factors limiting cells' growth, such as the immunological response of the organism to the tumor growth [1]. The highest carrying fitted capacity K is observed in "U-shape" category. It emphasizes that the carrying capacity does not affect the form of dynamics, because the tumor does not grow to the size, at which it would slow its growth rate. In some cases from this category the obtained carrying capacity was at the maximum value possible in the coding environment (10^{45}). That may indicate the absence of the carrying capacity and, thus, exponential growth of the cancer populations. In the "Growth" category the carrying capacity values are also elevated. This way the model reflects that the tumor has sufficient resources and space for the growth. In "Delayed response" and "Decline" categories K is significantly lower, indicating restrictions of the limited space and resources on the tumor.

The distributions of parameters in the "Delayed response" and the "Decline" categories are similar (Fig S4.1), as the model does not capture the initial increase in the size and instead describes the "Delayed response" trend as a monotonic decline. In the "Decline" category the growth rate ρ is lower and the treatment sensitivity λ is higher than in all others categories, except for the "Delayed response". This indicates that the tumors from "Decline" and "Delayed response" categories grow slowly in the absence of drug and have strong response to the applied treatment.

References

1. Thorsson V, Gibbs DL, Brown SD, Wolf D, Bortone DS. The Immune Landscape of Cancer. Immunity. 2018;48(4):812–830. doi:10.1016/j.immuni.2018.03.023.
