# Supplementary material for: Validation of polymorphic Gompertzian model of cancer through in vitro and in vivo data
Source: PLoS One. 2025 Jan 9;20(1):e0310844. doi: 10.1371/journal.pone.0310844 (PMC11717199; doi:10.1371/journal.pone.0310844)
Supplement: S5 Appendix — (PDF) [file pone.0310844.s005.pdf]

S5 Appendix.
Exponential growth and decay trends in the polymorphic Gompertzian model

When the polymorphic Gompertzian model was fitted to *in vitro* data, we observed a sudden "jump" in the obtained carrying capacity values (Fig 3 in main text). In several cases with higher proportions of sensitive cells ($p \geq 0.6$) and drug present, the value of the fitted carrying capacity K was close to 10^{45}, which is the maximum possible number in the coding environment.

After conducting additional tests, we established that the optimization procedure attempts to set the carrying capacity to the maximum possible value in the given search space. We hypothesize that this way the model indicates the absence of the carrying capacity and, hence, exponential growth of the resistant population and exponential decay of the sensitive population (as in the cases with drug present, the sensitive population declines).

As the model with shared growth rates fits the larger population better, the absence of the carrying capacity is likely attributed to the exponential decrease of the sensitive population. It is also justified by the fact that for some wells that contained only the sensitive population, the obtained carrying capacity was also at the highest possible number in the coding environment. If the resistant population is present in the well with the high K, it is small and, thus, has enough space and resources to grow. It is not clear whether the growth is exponential or logistic at the initial stage of increasing growth.

The growth can be both exponential or logistic (at the initial stage), as both cases can be fitted well with the polymorphic Gompertzian model with the high carrying capacity. The extension of the model with the separate growth rates and/or separate contributions to the carrying capacity for the sensitive and resistant populations can allow fitting trends in both populations better and assess the reasons for the switch from Gompertzian growth to exponential.

Carrying capacity values equal to the maximum number in the coding environment were also observed in several *in vivo* cases. Measurements in such cases coincide with exponential growth, which the model accurately fits. These cases require special attention, as the presence of the carrying capacity and/or competition between treatment-sensitive and treatment-resistant populations lead to better outcomes of evolutionary therapy, which aims to anticipate and steer the evolution of resistance in the cancer population. Thus, patients with exponential tumor growth would likely benefit only little from the adaptive therapy. However, for the majority of the patients, the carrying capacity is finite, and according to Viossat and Noble's theoretical conclusions, they would benefit more from the containment approach than from the MTD approach in terms of time to progression [1].

References

1. Viossat Y, Noble R. A theoretical analysis of tumour containment. Nature Ecology & Evolution. 2021;5(6):826–835. doi:10.1038/s41559-021-01428-w.
